# Rapid identification and recovery of ENU-induced mutations with next-generation sequencing and Paired-End Low-Error analysis

**DOI:** 10.1186/s12864-015-1263-4

**Published:** 2015-02-14

**Authors:** Luyuan Pan, Arish N Shah, Ian G Phelps, Dan Doherty, Eric A Johnson, Cecilia B Moens

**Affiliations:** Division of Basic Sciences, Fred Hutchinson Cancer Research Center, 1100 Fairview Ave. N., Seattle, WA USA; Department of Pediatrics, Division of Genetic Medicine, University of Washington, Seattle, WA USA; Institute of Molecular Biology, University of Oregon, Eugene, OR USA; Biology Department, University of Washington, Seattle, WA USA; Current Address: China Zebrafish Resource Center, Institute of Hydrobiology CAS, 430072 Wuhan, China

**Keywords:** TILLING, Zebrafish, Next-generation sequencing, PELE analysis, Rare mutation detection

## Abstract

**Background:**

Targeting Induced Local Lesions IN Genomes (TILLING) is a reverse genetics approach to directly identify point mutations in specific genes of interest in genomic DNA from a large chemically mutagenized population. Classical TILLING processes, based on enzymatic detection of mutations in heteroduplex PCR amplicons, are slow and labor intensive.

**Results:**

Here we describe a new TILLING strategy in zebrafish using direct next generation sequencing (NGS) of 250bp amplicons followed by Paired-End Low-Error (PELE) sequence analysis. By pooling a genomic DNA library made from over 9,000 N-ethyl-N-nitrosourea (ENU) mutagenized F1 fish into 32 equal pools of 288 fish, each with a unique Illumina barcode, we reduce the complexity of the template to a level at which we can detect mutations that occur in a single heterozygous fish in the entire library. MiSeq sequencing generates 250 base-pair overlapping paired-end reads, and PELE analysis aligns the overlapping sequences to each other and filters out any imperfect matches, thereby eliminating variants introduced during the sequencing process. We find that this filtering step reduces the number of false positive calls 50-fold without loss of true variant calls. After PELE we were able to validate 61.5% of the mutant calls that occurred at a frequency between 1 mutant call:100 wildtype calls and 1 mutant call:1000 wildtype calls in a pool of 288 fish. We then use high-resolution melt analysis to identify the single heterozygous mutation carrier in the 288-fish pool in which the mutation was identified.

**Conclusions:**

Using this NGS-TILLING protocol we validated 28 nonsense or splice site mutations in 20 genes, at a two-fold higher efficiency than using traditional Cel1 screening. We conclude that this approach significantly increases screening efficiency and accuracy at reduced cost and can be applied in a wide range of organisms.

**Electronic supplementary material:**

The online version of this article (doi:10.1186/s12864-015-1263-4) contains supplementary material, which is available to authorized users.

## Background

Obtaining stable mutant strains with mutations in high-priority genes is essential for a mechanistic understanding of biological processes. Over the last decade, with the increasing knowledge from whole genome sequencing, reverse genetic approaches are playing more and more important roles in providing genetic loss-of-function tools to the research community. TILLING (Targeting Induced Local Lesions IN Genomes) involves identifying and recovering rare mutant alleles in specific genes of interest from a large library of randomly mutagenized individuals, and is one of the most widely used reverse genetic techniques.

First developed in *Arabidopsis* in 2000 [1], TILLING has been applied to a range of plant and animal species [2-16]. The classical TILLING process involves PCR amplification of a specific target from the entire mutagenized library with fluorescent labeled primers and CEL1 enzyme digestion of the resulting PCR amplicons to cut any heteroduplexes caused by the presence of induced mutations that occur only once in the entire library. Full-length and rare cleaved fragments are detected by Li-Cor gel analysis, and point mutations are confirmed by Sanger sequencing [17-19]. Although this process has proven effective, it is limited to screening a single target at a time and its poor sensitivity only allows a low level of library pooling. Thus it is both labor-intensive and time-consuming. In addition, mutation detection relies on Li-Cor gel imaging which constrains the fragments that can be screened to 750–1200 bp, a size that is frequently incompatible with intron-exon structure. Furthermore, the approach is limited by the intrinsic specificity of the CEL1 endonuclease and is influenced by the level of pre-existing polymorphism in target genes.

We established an ENU-mutagenized library of 9,024 F1 male zebrafish, each with a unique set of ~5,000 ENU-induced heterozygous mutations and have used CEL1-based TILLING to identify and recover deleterious mutations from this library in over 50 genes [16]. In recent years, however, Next-Generation Sequencing (NGS) has provided the capability to process multiple TILLING targets at the same time. The general strategy for NGS-TILLING is to amplify multiple target exons from pools of template DNAs, and then to pool and barcode all of the targets from a single library pool for sequencing. To date, two groups have applied NGS to TILLING [20-22]. However in order to detect mutations over background PCR and sequencing errors, template pooling was limited to 96 individuals and the entire library was limited to fewer than 1,000 individuals. Furthermore, shearing of the PCR amplicons in preparation for Illumina sequencing resulted in uneven sequence coverage and thus incomplete screening of target fragments.

Here we introduce a new NGS-TILLING strategy that allows us to screen up to 30 PCR amplicons at a time in a library of over 9,000 mutagenized individuals with high efficiency and accuracy. Our method involves three innovations: 1) Rather than using a complex pooling strategy that triangulates on one or a few potentially mutant individuals [20,22,23], we simply use Illumina sequencing to identify mutations in a pool of 288 individuals under a single barcode; we then deconvolve that pool using high resolution melt (HRM) analysis; 2) Rather than amplifying large genomic fragments and shearing them to generate short overlapping fragments appropriate for Illumina HiSeq, a process which is time-consuming and yields uneven sequence coverage, we amplify 250 bp fragments corresponding to exons of interest and sequence them directly using the Illumina MiSeq platform; 3) In order to eliminate sequencing error as a source of false positives, we do paired-end sequencing of the entire 250 bp amplicon, align the two sequences from each cluster and reject any overlapped reads with less than perfect sequence identity. This “Paired-End Low-Error” analysis is described elsewhere (E. Johnson, manuscript in preparation) but is similar in principle to the method recently described [24]. We have confirmed that our NGS-TILLING method is able to identify known mutations previously identified by CEL1 TILLING, and furthermore demonstrate that it can identify mutations that were previously not found with CEL1. We have gone on to test our method with 109 target fragments from 32 zebrafish genes, and identified 28 nonsense mutations in 20 of these genes with an acceptable false-positive rate of 38.5%. While being developed for mutation identification in the zebrafish, our approach is applicable to any species that is amenable to chemical mutagenesis.

## Results

### Library pooling and fragment preparation

**Figure 1:**
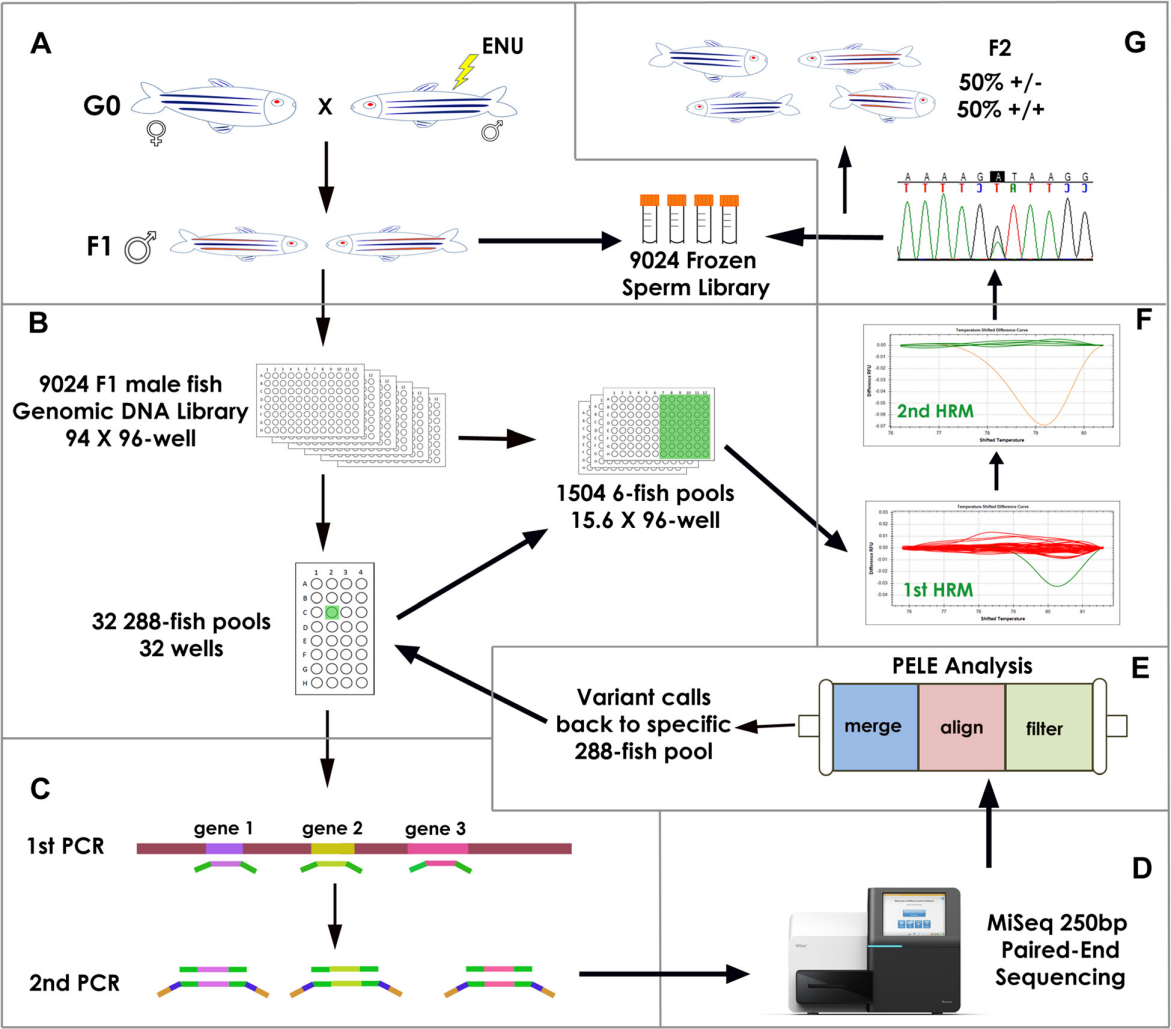
**NGS-TILLING process. A**: A long-term resource for many TILLING screens consisting of a genomic DNA sample and a corresponding cryopreserved sperm sample was prepared from 9,024 F1 ENU-mutagenized male zebrafish. **B**: Library Pooling. Normalized genomic DNA (gDNA) was pooled twice: first, gDNA from 6 fish was pooled together to make 1,504 6-fish pools in 16 96-well plates. These six-fish pools will be used for HRM identification of carrier fish (step **F**). Second, groups of 48 6-fish pools were pooled together into 288-fish pools (a total of 32 288-fish pools). **C**: Target Preparation. gDNA from 288-fish pools was used as a template for PCR amplification of ~250 bp fragments corresponding to exons of genes of interest using gene-specific primers with P5/P7 SEQ tails (green). After normalization, amplicons from each 288-fish pool were combined and used as template for a brief second PCR that added Nextera index sequences (blue) and Illumina P5/P7 sequences (yellow). **D**: Sequencing: All amplicons from the entire library were combined and sequenced (Illumina MiSeq platform), generating fully overlapping 250 bp paired-end sequences. **E**: Data Analysis. Sequence analysis using PELE and PoDATA identified rare deleterious variants (occurring in 1/100 to 1/1000 reads) in single 288-fish pools. **F**: Deconvolution. A fragment centered on a putative variant call was amplified from each of the 48-six-fish pools used to make up the 288-fish pool in which that variant was detected, and was subjected to High Resolution Melt (HRM) Analysis. Then HRM of the six individual fish in the six-fish pool that showed distinct melting kinetics identified the individual carrier. **G**: Mutant Recovery. Finally, the presence of the variant identified by PELE and PoDATA was confirmed in that fish by Sanger sequencing. F2 heterozygotes were generated by in vitro fertilization of WT eggs with the corresponding cryopreserved sperm sample.

For any TILLING approach, a large population of mutagenized individuals is required. We generated a library of 9024 ENU-mutagenized F1 male fish by treating wild type (WT) adult male fish with ENU to mutagenize their spermatogonia, crossing them with WT females, and raising F1 male progeny to adulthood [16]. Each F1 fish carries a unique set of heterozygous ENU-induced mutations, so any given mutation occurs only once in the entire library, i.e. at a ratio of 1 mutant: 18,048 WT alleles. Sperm from these males was cryopreserved and their carcasses were used for the preparation of genomic DNA as described [16] (Figure 1A).

This genomic DNA library from 9024 fish was normalized and subdivided into pools of 288 fish each (30 288-fish pools plus 2 192-fish pools) for NGS-TILLING. The 288-fish pool size was determined empirically to be the largest number of fish that allowed us to unambiguously identify induced mutations, which are expected to occur in a single pool at a frequency of 1:576 alleles, over mutations introduced by the PCR or sequencing steps (Figure 1B). We have also sequenced pools of 576 fish (1152 alleles) and were able to identify known variants but this incurred a 2-fold higher false positive rate to identify most of the known variants. Therefore, we chose to screen pools of 288 fish.

We amplified and directly sequenced the largest fragments possible using available NGS technology, without shearing or otherwise fragmenting the template. The MiSeq platform generates 25 million 250 bp paired-end sequencing reads (500 cycle version 2 reagent kit). We estimated, given this capability, that we could sequence 25 250 bp fragments in two directions from each of the ~18,000 haploid genomes in the library at sufficient coverage to detect multiple reads of a mutant allele that appears only once in a single 288-fish library pool. We chose 250 bp fragments rather than 500 bp fragments so that the MiSeq paired-end reads would be fully overlapping (see Sequence analysis section below).

The detailed protocol for target fragment preparation is provided (Additional file 1). Briefly, we used gene-specific primers to amplify 210- to 270 bp fragments corresponding to conserved exons in genes of interest. The genes were identified by members of the zebrafish community as being of high biomedical interest, and were submitted via an online request site (https://webapps.fhcrc.org/science/tilling/) (Additional file 2, Table 1). For each gene of interest, multiple exons were selected as target fragments. Wherever possible, we used CODDLE (Codons Optimized to Discover Deleterious LEsions) [25] to identify exons in which ENU has the highest likelihood of generating nonsense mutations. A first round of PCR (30 cycles) amplified target fragments from genomic DNA using a pair of gene-specific primers with Illumina P5/P7 SEQ tails. Equal amounts of each of the 25 gene-specific PCR products from each 288-fish pool were combined and briefly amplified (5 cycles) using Illumina Nextera index primers to add a pair of specific Illumina indices and P5/P7 tail to the amplicons from each pool (Figure 1C). Finally, the indexed fragments from all 32 pools were pooled for loading onto an Illumina MiSeq desktop sequencing machine using the MiSeq v2 Reagent Kit per manufacturers instructions.

**Table 1:** **Summary of NGS-TILLING findings**

**Genes**	**Fragment screened**	**Exon screened**	**Total target size**	**Coding size**	**Variants tested**	**Variant confirmed**	**Deleterious mutations**
lef1	1	1	258	163	19	11	0
amer1	4	1	1007	828	3	3	1
atoh1b	2	1	510	442	2	1	0
col4a3	3	5	804	417	3	0	0
col4a4	5	6	1263	746	3	1	1
col4a6	4	4	978	623	1	1	1
cspp1b	3	3	713	450	5	3	3
eml1	4	4	974	578	2	2	1
Exosc3	3	3	728	514	2	0	0
flt1	4	4	1004	576	3	3	1
FUS	3	3	809	578	0	0	0
hif1ab	5	5	1305	749	4	1	1
irf6	2	2	521	417	9	1	0
kif7	7	7	1846	1280	3	2	2
lycat	2	2	492	352	1	0	0
map3k12	6	6	1492	1019	3	1	1
maza	3	3	723	546	0	0	0
mllt4	6	6	1468	1097	1	0	0
myo10l1	2	2	516	335	3	1	1
nbeab	5	5	1239	809	7	4	2
oit3	4	3	1018	652	3	1	1
orc1	3	3	786	556	3	3	3
pax7a	1	1	262	222	2	0	0
pkd2l1	2	2	538	420	1	1	1
ppp4ca	3	4	781	404	1	1	1
prickle1a	3	3	715	447	0	0	0
ptk7	4	4	1020	767	3	2	1
rbfox1l	2	2	536	417	1	0	0
ryk	3	3	738	415	1	1	2
slc25a21	4	4	951	319	5	2	2
sox19b	3	1	771	601	1	1	1
tnfsf10	3	3	770	491	1	1	1
Total	109	106	27536	18230	96	48	28

109 target fragments, from 106 exons in 32 genes screened by NGS-TILLING. The total amount of genomic DNA screened was 27.5 Kb in each of 9,024 fish, corresponding to almost 250 Mb of sequence. Of this 27.5 Kb, 18.2 Kb coding sequence. 28 deleterious mutations (nonsense and splice site mutations) were found in 20 genes out of 32 genes.

### MiSeq sequencing

**Figure 2:**
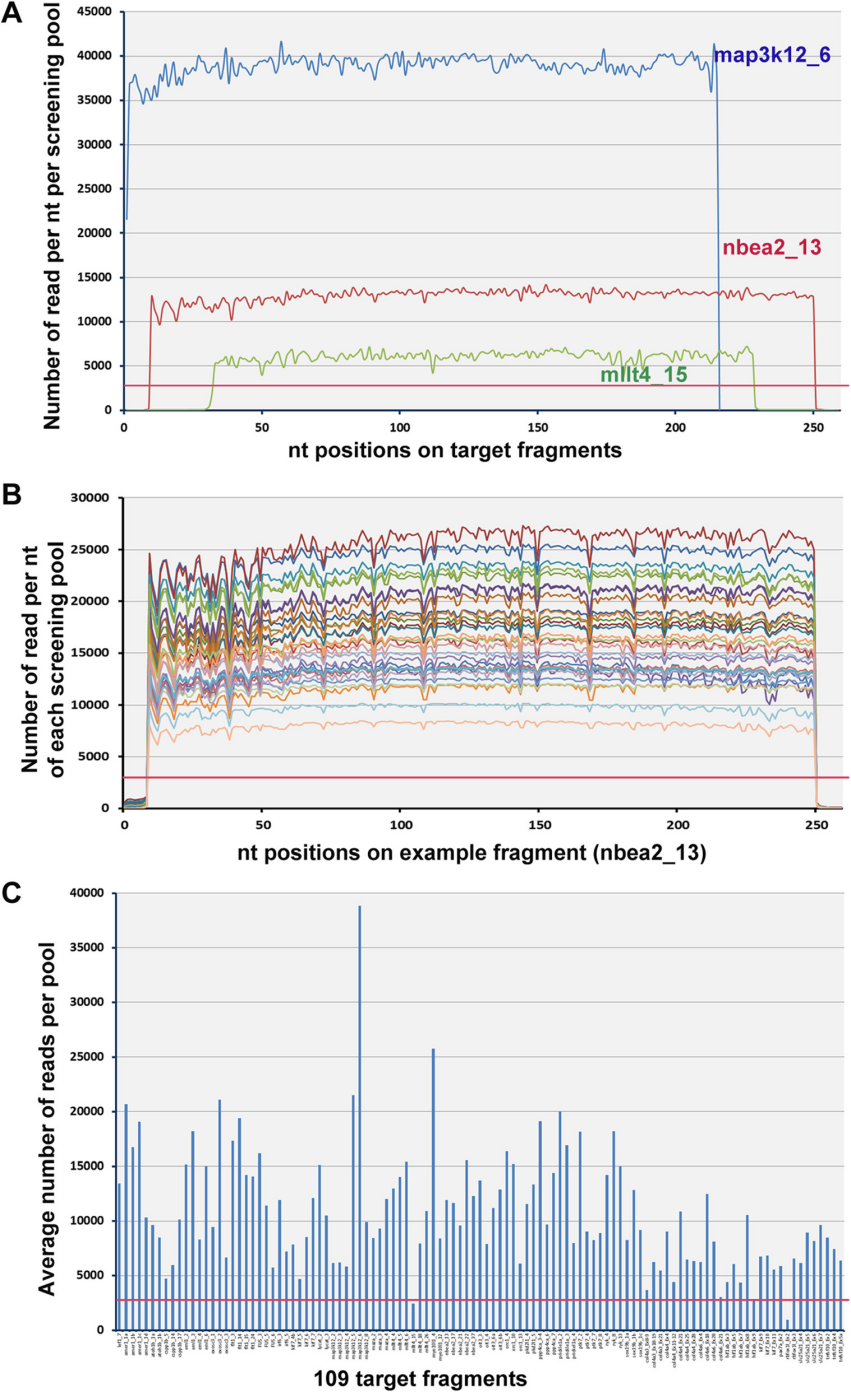
**Sequence coverage from direct MiSeq PE250 sequencing. A**: Direct sequencing of target amplicons without shearing produces homogeneous sequence coverage. Representative average read coverage of three amplicons under a single barcode with high (blue), medium (red) and low (green) coverage. Even the poorest sequence coverage exceeds the minimum coverage needed to accurately identify mutations in our 288-fish pools (red line). **B**: Amplicon coverage across all library pools. Different colors represent the same amplicon from each of the 32 pools in the library. **C**: Variable coverage of different fragments. Each bar represents the average coverage across the entire library of a single randomly selected nucleotide in each of 109 fragments screened. 107 of 109 fragments exceeded the minimum coverage needed to accurately identify mutations in our 288-fish pools (red line).

Using the approach outlined above we screened our 9,024-fish library for 109 target fragments from 32 genes (a total of 27.5 Kb) in five MiSeq runs (Table 1, Additional file 2). In each of the sequencing runs we loaded 15–20 pM sample and obtained an average cluster density of 802/mm^2^ and an average of 85% of clusters passing the quality filter (>Q30 ratio), corresponding to an average of 3.7 Gb of raw sequence (14.8M 250 bp reads) per run. In the first step of the data analysis, ~15% of the raw data (0.7 Gb) was identified as sequencing error and discarded (see next section for details). The rest of the data was processed for alignment of each target fragment. Coverage of each fragment was very even within a pool, with read depth varying only 2-10% across the length of fragments (Figure 2A), while read depth for different fragments varied as much as 10-fold (Figure 2A, C) and read depth for the same fragment in different pools varied as much as 3-fold (Figure 2B).

We chose 2,880 reads as the minimum number of reads at each position in each pool for screening, as this corresponds to 5 reads per allele assuming equal amplification of each allele in the pool: $\left(\frac{2880\  reads/ pool}{288\  fish/ pool \times 2\  allele s/ fish}=5\  reads/ allele\right)$ (red line in Figure 2A,B,C). At this level, a mutant allele that is present in the heterozygous condition in a single fish in the pool should be detected at least once. 107 out of 109 target fragments exceeded this minimum, with an average read coverage of 10,854 ± 5,549 per pool (S.D.; Additional file 3). The remaining two fragments were analyzed (see below) but were not considered fully screened.

### Sequence analysis and filtering

We used Paired-End Low-Error (PELE) analysis to detect rare ENU-induced mutations in our MiSeq dataset (E. Johnson, manuscript in preparation). Briefly, PELE concatenates and processes data from several sequence analysis programs. First, it merges the two overlapping sequences generated by paired-end sequencing of each cluster using the SeqPrep program (https://github.com/jstjohn/SeqPrep) and filters out any imperfectly aligned sequences, thereby eliminating errors that occurred during the sequencing process that would occur in one but not both paired-end reads.

Next, PELE aligns the reads that passed the first filter to our reference sequences using the Novoalign (http://novocraft.com) software and detects all single-base variants using SAMtools (http://samtools.sourceforge.net/) [26]. For each variant, PELE assesses its frequency (F) in the pool in which it occurred as:

$$ F=\frac{number\  of\  variant\  reads}{total\  number\  of\  reads\  at\  that\  position\  in\  that\  pool}. $$

**Figure 3:**
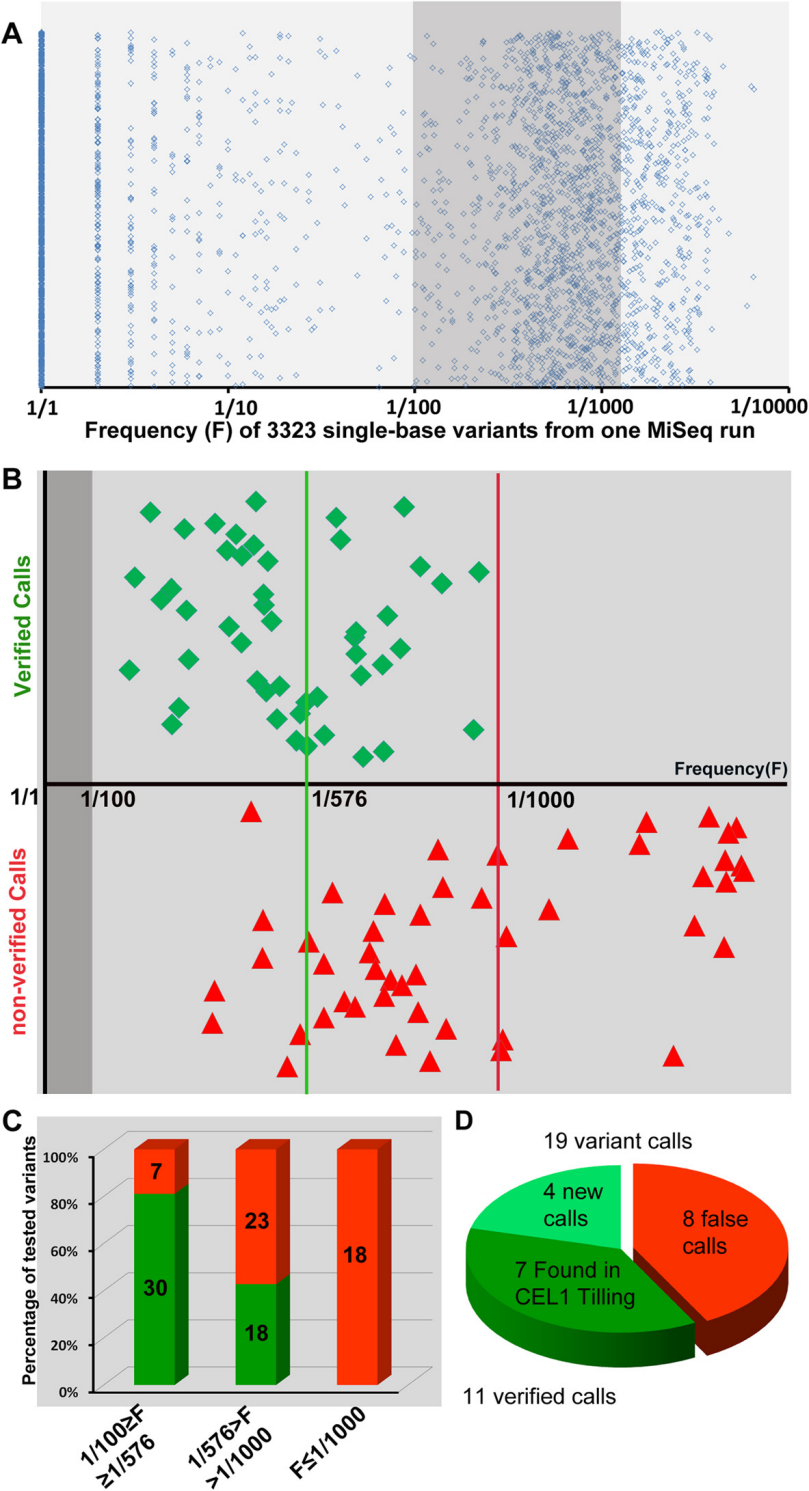
**Mutation distribution and validation. A**: Frequency distribution of all variants from one MiSeq run after PELE analysis. 3323 variant calls were made by PELE analysis, ranging from 1/1 to 1/6513. We selected 96 putative variants that occurred at a frequency between 1:100 and 1:1500 for validation (shadowed area). **B**: Frequency of 96 variants chosen for validation using PoDATA. Green diamonds are variants that were confirmed as being genuine ENU-induced mutations; red triangles are variants that failed to be confirmed (false positives). The X axis shows the frequency with which each variant appeared within its pool. Variants in the dark grey area (F > 1/100) were filtered out as pre-existing polymorphisms. The vertical green line is the theoretical frequency for a unique ENU-induced mutation within a pool (1/576 alleles). The red line is the upper bound of verified calls (1/1000). **C**: Summary data in Figure 3B. Green bars represent confirmed variants, red bars are false positives in each of three frequency bins. **D**: Comparison of CEL1 vs NGS-TILLING using a control fragment. NGS-TILLING detected 19 variants of which 11 were verified (green) including all 7 mutations found previously using CEL1-based TILLING (darker green).

Using PELE, in each MiSeq run we identified 3000–5000 variants at frequencies ranging from F = 1/1 to F = 1/6573 (Figure 3A). Single base variants have three possible origins: 1) they may be the ENU-induced mutations we wish to identify; 2) they may be polymorphisms that existed in the parental fish prior to mutagenesis; 3) they may have been introduced during the PCR amplification of target fragments. An ENU-induced mutation is expected to exist in a single F1 fish in the library in the heterozygous condition, so it should appear in a single 288-fish pool at F≅1/576 assuming equal amplification of all alleles in a pool. Pre-existing polymorphisms are expected to be much more frequent, since the library was made from less than 50 closely related ENU-mutagenized G_0_ fish [16]. We assume that pre-existing polymorphisms occur at a frequency of F > 1/100, so we excluded from further analysis any variants occurring at F > 1/100.

Variants introduced by PCR can appear at any frequency depending on when in the amplification process they occurred, but even if they arose very early in the amplification process their frequency is not anticipated to be higher than that of the ENU-induced mutations in the template genomic DNA. We determined the frequency threshold at which variants introduced in the amplification process outnumber ENU-induced variants empirically, by attempting to validate 96 sequence variants that occurred at a frequency between 1 (mutant allele):100 (wildtype alleles) and 1:1500 (Figure 3B, Additional file 4. For the validation approach see “Mutation verification” section below). The 96 variants we attempted to validate were chosen from among all the variants in all 109 fragments that were called based on their high likelihood of causing loss-of-function phenotypes. To facilitate selection of potentially deleterious variants, we developed a program, “Predictor of Deleterious Alleles in Target Amplicons” (PoDATA) (Additional file 5, Additional file 6) that predicts all possible single nucleotide substitutions in target fragments that can cause a nonsense mutation or change an RNA splice site, and then flags NGS-TILLING variants that fall into this data set.

Of these 96 variants, we confirmed 30/37 (81%) of variants that were called at a mutant/wildtype ratio between 1/100 > F > 1/576. We also confirmed 18/41 (44%) of variants that were called between 1/576 > F > 1/1000 (Figure 3C; see “Mutant verification and recovery” section below). None (0/18) of the variants that appeared at lower frequencies (F < 1/1000) could be validated; we conclude that these represent errors introduced by PCR. Thus by setting our frequency filters at 1/100 > F > 1/1000 we were able to validate 61.5% (48/78) of putative mutant calls (Figure 3B, C); the false positive rate is thus 100–61.5% = 38.5%. These validated mutations included 28 deleterious mutations (nonsense and splice site mutations) in 20 genes; an efficiency of 1 deleterious mutation per 650 bp of coding sequence. This compares favorably with an efficiency of 1 nonsense mutation per 1400 bp of coding sequence screened in the same library with CEL1 TILLING [16].

Importantly, in the 109 target fragments we screened, we included one fragment that, using the CEL1 TILLNG methodology, we had previously identified 7 ENU-induced mutations in our library (lef1_ex7, Table 1 and Additional file 2). Using the NGS-TILLING approach described above, we made 19 variant calls in this fragment, and verified 11 of them, including all 7 mutations previously identified by CEL1 TILLING plus 4 new mutations (Figure 3D). Thus based on our overall nonsense mutation recovery rate and this direct comparison, NGS-TILLING is significantly more effective at identifying rare mutations than CEL1 TILLING.

### Validation of PELE method for identifying rare mutations

The merging of overlapping paired-end sequences with PELE identified 14.5 ± 4.8% (SD) of the raw data as error and discarded it. To determine whether this step was necessary for eliminating false positives, we compared the number of variants identified with and without this merging step. In one MiSeq run with a total target size of 5.5 Kb, PELE analysis identified a total of 1,115 variants in the range of 1/100 > F > 1/1000. However, direct alignment of raw sequences against reference sequences without merging identified 56,467 variant calls - 50.6 times more calls in the same F range. We attempted to validate 56 of the extra calls that were generated without paired-end sequence merging (see “Mutation verification” section below) and failed to validate any of them. These data demonstrate that PELE analysis efficiently reduces noise of sequencing error from real variants: at the cost of losing ~15% of the raw sequence data, PELE analysis filtered 98% of the noise generated in the sequencing process.

### Mutation verification and recovery

**Figure 4:**
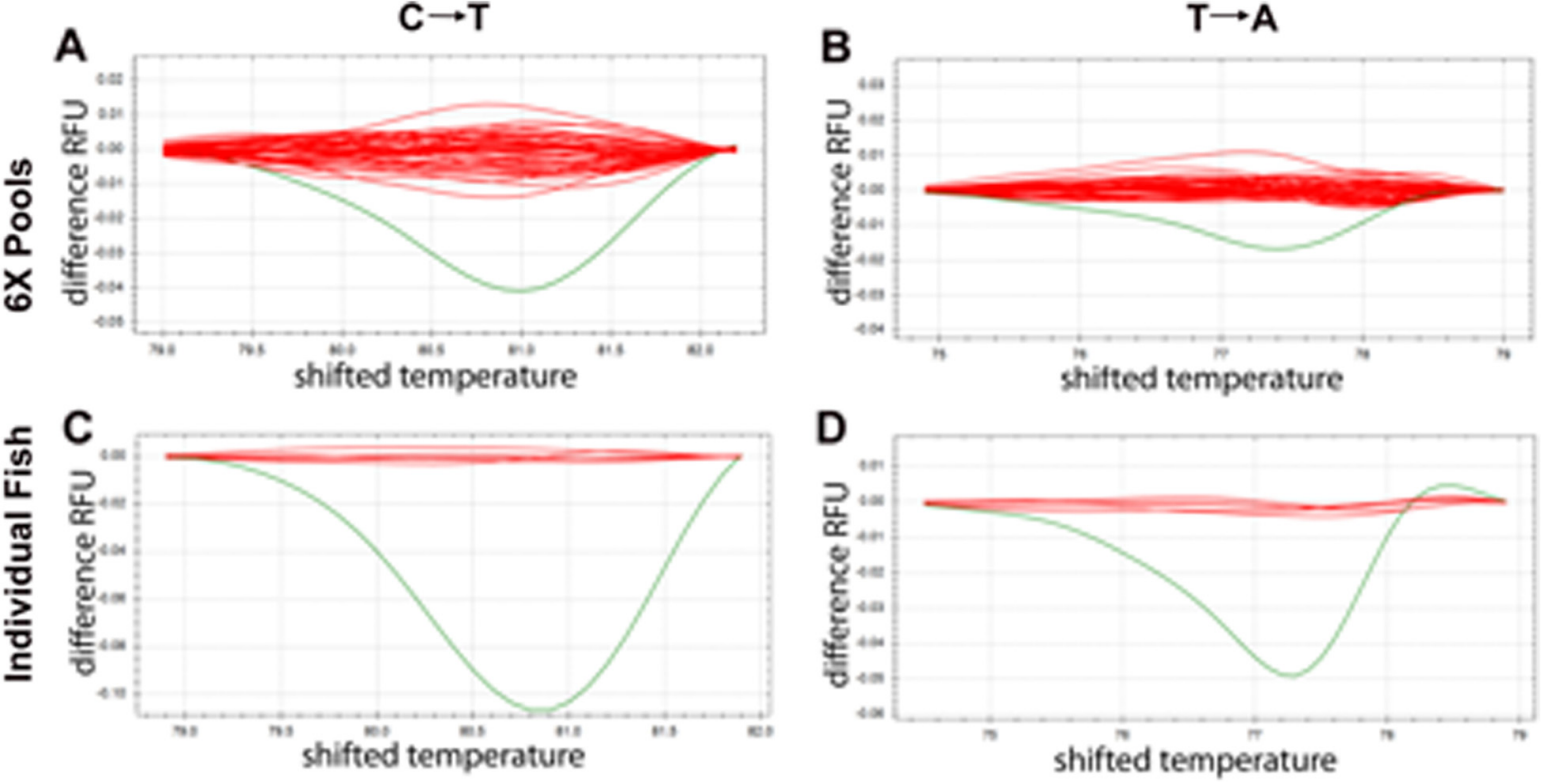
**Variant validation using High Resolution Melt analysis. A**, **B**: HRM on 6-fish pools identifies a single pool (green line) with distinct denaturation kinetics. Each line represents a single six-fish pool. The X axis is temperature and Y axis is the fluorescent difference compared to the wildtype melt curve. C → T mutations **(A)** cause a larger temperature shift than T → A mutations **(B)**. **C**, **D**: HRM on single fish of the six-fish pool identified above. Each line represents one fish (2 alleles).

The NGS-TILLING method described above identifies a pool of 288 fish that includes a single fish with a specific single heterozygous nucleotide change. Since at this point we knew the exact sequence of the variant, we reasoned that we could use a standard genotyping approach to locate that fish within the pool. High Resolution Melt (HRM) analysis detects mutations in double stranded PCR amplicons due to their different disassociation kinetics at increasing temperatures [27]. We determined that a known single nucleotide mutation is detectable by HRM when present as one allele in 24 (1 heterozygous carrier in 12 fish) and can be robustly detected at a ratio of 1:12 alleles (Additional file 7). Accordingly, we re-pooled the genomic DNA library into 6-fish pools such that each 288-fish pool comprised 48 6-fold pools, and amplified ~100 bp fragments centered on the variants identified by NGS-TILLING from each of the 6-fold pools from the 288-fish pool in which that mutation was found (Figure 1F). HRM of these fragments efficiently identifies the 6-fold pool containing the mutant fish (Figure 4A, B) and a second round of HRM of these six fish identifies the mutant individual, which was confirmed by Sanger sequencing and recovered from cryopreserved sperm (Figure 4C, D; Figure 1G). We note that mutations that alter base pairing (C:G ↔ A:T), which account for ~75% of mutations generated by ENU, are more easily detected by HRM than mutations that maintain nucleotide valence (A:T ↔ T:A; Figure 4). In some cases, difficulty of detecting A:T ↔ T:A mutations in 6-fold pools necessitated screening all of the 288 fish in a pool individually by HRM.

## Discussion

TILLING is one of the most widely used reverse genetic approaches to detect single base pair mutations in specific genes of interest in mutagenized or natural genomes [2,4,6,10-12,14,15,18,21,28-40]. The low throughput of the CEl1-based TILLING strategy [19] has motivated several groups to develop alternative methodologies including direct mutation detection using HRM [37,41,42], or massively parallel (next-generation) sequencing (NGS) of specific targets [20-23,43]. The approach we describe here improves on these by allowing higher throughput and better target coverage at reduced cost and increased efficiency. In our hands, one person can screen 25 250-bp fragments on our library of 9,024 individuals, from primer design to mutant recovery in 4 weeks.

The NGS platform offers the ability to detect rare mutations simultaneously in multiple target genes in a large mutagenized population, ideally, a population large enough to include at least one loss-of-function mutation in every gene in the genome. The first major challenge is in detecting a specific mutation, which is expected to occur only once in the entire population, over a background of mutations introduced during PCR preparation and sequencing of target fragments. NGS is associated with a ~1% error rate [44], which in previous NGS-TILLING attempts has limited the size of the pool of individuals under a single barcode to 96 [20,21] and the entire library to 384 [21] or 768 [20] individuals. By merging the two fully overlapped sequences generated by paired-end sequencing of 250 bp amplicons and eliminating any imperfectly aligned reads, we have effectively eliminated sequencing error as a source of noise (98%) in our analysis. This has allowed us to increase the pool size to 288 heterozygous fish under a single barcode.

A second source of false positives is mutations introduced during the preparation of target amplicons for sequencing. These PCR-introduced mutations are expected occur at a lower frequency than the ENU-induced mutations present in the template (1/576 alleles in our pools). By eliminating from our analysis variants called at a frequency lower than 1/1000 we were able to confirm more than 60% of variant calls in our ENU-mutagenized library without discarding any genuine ENU-induced variants. The remaining false positives are likely to represent variants introduced in the early cycles of target amplicon preparation.

A number of bi-directional and multi-directional pooling strategies have been proposed that allow the unique identification of rare mutant individuals directly by TILLING [20-23,43]. The large number of pools required by these strategies significantly increases the amount of PCR involved in preparing targets and the number of barcodes required, while limiting the total number of alleles that can be screened: in these strategies, over 100 barcodes are needed to screen only 2000 individuals. We reasoned that once a variant has been identified in a single one-dimensional pool by NGS-TILLING, that pool can be deconvolved secondarily using a simple allele-specific genotyping method such as HRM. This, combined with our PELE analysis that identifies and eliminates errors introduced during sequencing, enabled us to screen a library of over 9,000 individuals using only 32 barcodes. A further efficiency we have introduced is the use of the relatively long sequencing runs available on the MiSeq platform, which eliminates the need for fragmentation of PCR amplicons, a process that has been shown to introduce uneven sequence coverage of PCR-amplified target sequences [20,21,23] (our own data not shown).

Sensitive detection of very rare mutations is needed not only for TILLING but increasingly for other areas of biomedical research where complex mixtures of cells with different genotypes, as in cancer and somatic mosaic disease, are studied [45]. To discriminate genuine mutations from variants introduced during PCR or sequencing, a variety efforts have been made from sample processing to data analysis [46-50]. These methods use deep sequencing of the samples tagged with long and redundant barcodes, random barcodes, or endogenous random shear points. While all of them significantly improved signal-to-noise ratio, they do not apply well to TILLING because of their high cost, complex of PCR and ligation strategies, and/or their inability to track a mutation back to a specific mutation carrier. Our methodology identifies one induced point mutation in more than 18,000 alleles, and distinguishes it from pre-existing polymorphisms and errors introduced during PCR and sequencing with just 32 pairs of commercially available barcodes. The 96-index system currently available from Illumina could be used to expand TILLING capability to a library of 28,000 heterozygous individuals. Furthermore, the new MiSeq system v.3 generates 40M 300-bp reads, doubling the amount of target sequence that can be screened in a single run. Importantly, every aspect of the methodology that we have developed for NGS-TILLING of zebrafish can be applied to other organisms for rare mutation detection and recovery.

## Conclusions

TILLING is a widely used technique to screen for rare mutations in large populations. In this work, we present a simplified and rapid TILLING approach using direct Illumina MiSeq sequencing of 250 bp target amplicons, PELE data analysis to remove false positive mutant calls and HRM to identify specific mutant carriers within our library. Our new NGS-TILLING system is able to detect unique point mutations among more than 18,000 alleles using only 32 pairs of barcodes. We detect one strongly deleterious (nonsense or splice site) mutation per 650 bp screened in the library, with an acceptable false positive rate of 38.5%. In principle, our NGS-TILLING system can be expanded to detect a unique variant among 50,000 wildtype alleles and is directly applicable to any organism.

## Methods

### Cryopreserved sperm and genomic DNA libraries

The ENU mutagenesis and sperm cryopreservation approaches used for the preparation of our zebrafish TILLING library were previously described [16,51]. Carcasses were frozen until Genomic DNA was extracted using the DNeasy 96 Blood & Tissue Kit (Qiagen). DNA from 9024 fish was normalized to 10 ± 2 ng/μl, and stored in 94 96-well plates. Normalized DNA from 6 consecutive fish was pooled together to build 1504 6-fish pools, which were stored in 16 96-well plates. Finally, every half plate (48 wells) of 6-fish pools were combined to make 30 288-fish pools plus 2 192-fish pools. These 288-fish pools were used at 10 ng/μl as template DNA for PCR.

### TILLING target preparation

A more detailed protocol is provided in Additional file 1. We chose conserved exons toward the 5′ end of target genes for screening. We gave preference to >100 bp exons in which there was a high likelihood of ENU causing nonsense mutations. Gene-specific primers designed using Primer3 (v. 0.4.0) were tagged with P5/P7 SEQ tails:

Forward: 5′TCGTCGGCAGCGTCAGATGTGTATAAGAGACAG, Reverse: 5′GTCTCGTGGGCTCGGAGATGTGTATAAGAGACAG and PCR was carried out using Phusion high-fidelity PCR master mix (BioLabs). Each fragment was amplified separately from each 288-fish pool (i.e., for a single MiSeq run: 25 fragments × 32 288-fish pools = 800 PCR reactions). Each 10 μl, 30-cycle PCR reaction contains 5 μl Phusion mix, 1 μl (10 ng) genomic DNA from a 288-fish pool, 0.25 μl 5 μM primer mix, and 3.75 μl H_2_O. The optimal annealing temperature was determined for each primer pair in advance via a PCR gradient test.

We ran 4 reactions for each fragment on a SYBR Safe (Invitrogen) gel, and quantified the average yield of this fragment (GelDoc system, BioRad). 30 ng of each of the PCR products amplified from each 288-fish pool were pooled together and cleaned up using the DNA Clean and Concentrator kit (ZYMO). Cleaned up products were used as the template DNA for a brief (5 cycles) second PCR with Nextera index primers (Illumina) using HiFi HotStart ReadyMix PCR Kit (KAPA). Each 50 μl reaction contained H_2_O, 25 μl ReadyMix (KAPA Biosystems), 50 ng pooled PCR product from the first PCR, 5 μl outside primer mix (Forward: AATGATACGGCGACCACCGA, Reverse: CAAGCAGAAGACGGCATACGA) and 2.5 μl Nextera™ i7 primer, 2.5 μl Nextera™ i5 primer (Illumina), which contained Illumina indices and barcoded the PCR products from same 288-fish pool. PCR products were cleaned up with DNA Clean and Concentrator Kit (ZYMO), and yields were again run on SYBR Safe gel and quantified (GelDoc system). 30 ng of the indexed products from each 288-fish pool were pooled together so that the final mix consisted of all of the fragments from all of the pools. The concentration of final mix was accurately quantified using SYBR FAST Universal qPCR kit (KAPA). See Additional file 1 for details.

### MiSeq sequencing

Prepared target libraries were sequenced using Illumina’s MiSeq Desktop Sequencer. Briefly, the target library was denatured, diluted to 15pM, spiked with a premade PhiX control library at 5% (PhiX control v2, Illumina), loaded into a MiSeq v2 Reagent Kit (500 Cycles PE, Illumina). Sequencing generated paired-end (2 × 250 bp) dual-indexed (2 × 8 bp) reads. Following sequencing, reads were demultiplexed with the MiSeq Reporter software and store as FASTQs for downstream processing and analysis.

### PELE analysis

Using the PELE analysis method [E. Johnson, manuscript in preparation], we determined the frequency of variants existing in a pool of PCR amplicons. The method eliminates errors introduced during sequencing by generating fully overlapped paired-end reads and then merging them and eliminating any merged sequences that contain mismatches. Since the same sequencing error is not expected to occur in both paired-end reads, this PELE filter eliminates reads with errors that occurred during sequencing. To do this, raw paired-end reads are processed through SeqPrep, a program originally designed to merge paired-end Illumina reads that are overlapping into a single longer read (https://github.com/jstjohn/SeqPrep). In order to eliminate pairs of reads that do not match we set the minimum fraction of matching bases to overlap reads at 0.97. The now-merged reads are aligned to the reference sequence of the fragment using Novoalign V3.02.02 in a single-ended read fashion (http://novocraft.com). SAMtools V0.1.19 mpileup is then used, without probabilistic realignment, to determine the read coverage at each nucleotide for the four bases (http://samtools.sourceforge.net/) [26]. Based on this coverage, a frequency value for each variant is determined.

### Identification of mutant fish using High Resolution Melt analysis (HRM)

We identified the single mutation carrier in a 288-fish pool using two rounds of HRM. The first HRM used the 48 6-fish genomic DNA pools comprising that 288-fish as template. HRM primers were designed around the variant identified by PELE analysis, with amplicon sizes between 60 bp and 150 bp. The HRM reaction mix contained 10 μl 2XHRM mix (BioRad), 1 μl 5 μM primer mix, 1 μl (10 ng) 6-fish pooled template DNA, and 18 μl H_2_O. The second HRM used genomic DNA from the six individual fish in the six-fish pool where the mutation was detected. HRM was performed on a CFX Connect™ Real-Time PCR Detection System (BioRad), and results were analyzed by Precision Melt Analysis™ Software (BioRad). See Additional file 1 for details.

### Ethics statement

The work presented here did not involve human subjects, material or data. Zebrafish research is compliant with the American Veterinary Medicine Association Guidelines on the Care and Use of Aquatic Animals in Research, and with federal policy on the care and use of animals in research. It was approved by the Fred Hutchinson Cancer Research Center Institutional Animal Care and Use Committee (Protocol #1392) Supporting data is provided in this submission (see “ADDITIONAL INFORMATION” below).

## Additional files

Additional file 1:
**NGS-TILLING protocol.**
Additional file 2:
**Information about 109 target fragments.** 109 target fragments were listed by gene name and exon number. lef1_ex7 is the positive control fragment which was previously screened by CEL1-TILLING. Additional file 3:
**Sequencing coverage of all target fragments.** Row 1 identifies the 288-fish pool used for screening. Column A describes the 109 target fragments shown in Additional file 2. Numbers in each cell are the average read number per nucleotide. Additional file 4:
**Examples of variant calls.** PELE output of target fragment col4a6_Ex18. Row 1 is the 32 288-fish pools. Column B is the nucleotide position in the reference sequence. Column C is the variant possibilities in each nucleotide position: the first nucleotide is the reference sequence followed by three possible variants. Nucleotides in introns are in lower case and exonic nucleotides are capitalized. The numbers following the reference nucleotide are the total read number of this nucleotide in each pool. If a variant was detected, the absolute number of variant reads and the frequency is displayed (F = variant reads : total reads). For example at position 45, the reference sequence is c. In pool 66B, a variant A call was detected in 6 reads of a total of 2999 reads (greyed box), for an F = 1:498. A total of 55 variant calls were made in this example fragment. Additional file 5:
**Prediction of Deleterious Alleles in Target Amplicons (PoDATA) program.**
Additional file 6:
**PoDATA readme.**
Additional file 7:
**HRM Detection of Mutant Alleles at Various Mut:WT Ratios.** High Resolution Melt (HRM) Analysis Detection of Mutant Alleles at Various Mut:WT ratios. The mutant allele being tested here is *inka1af* ^*h326*^, a C > T mutation resulting in a nonsense mutation, R120X. HRM melt curves corresponding to wildtype (red; 0 mutant alleles), 12 animals (dark blue; 1 mutant allele in 24 alleles), 6 animals (light green; 1 in 12 alleles), 4 animals (pink; 1 in 8 alleles), 3 animals (light blue; 1 in 6 alleles), 1 animal (dark green; 1 in 2 alleles). Although the deflection due to the mutant allele is much more dramatic at lower ratios, it can still be detected at a 1:12 ratio (dark blue line).

## Abbreviations

TILLING: Targeting induced local lesions IN genomes
NGS: Next-generation sequencing
PELE: Pair-End Low-Error
ENU: N-ethyl-N-nitrosourea
WT: Wild type
HRM: High Resolution Melt
