# Supplementary material for: Rapid identification and recovery of ENU-induced mutations with next-generation sequencing and Paired-End Low-Error analysis
Source: BMC Genomics. 2015 Feb 14;16(1):83. doi: 10.1186/s12864-015-1263-4 (PMC4457992; doi:10.1186/s12864-015-1263-4)
Supplement: Additional file 1: — NGS-TILLING protocol. [file 12864_2015_1263_MOESM1_ESM.docx]

**Moens lab**

**NGS-TILLING Protocol**

1. DNA library and pooling
2. Target selection and primer design

Primer Test

Gel

1. 1^st^ PCR (Target specific PCR)

PCR all fragments from all pools (#Frag x #Pools)

Gel + Gel Quantify

Pool in the yellow direction

Cleanup

1. 2^nd^ PCR (Illumina indices PCR) and Pool

PCR to add indices to all pools (#Pools)

Gel

Cleanup

Spectrophotometer quantify

Pool in the green direction

1.
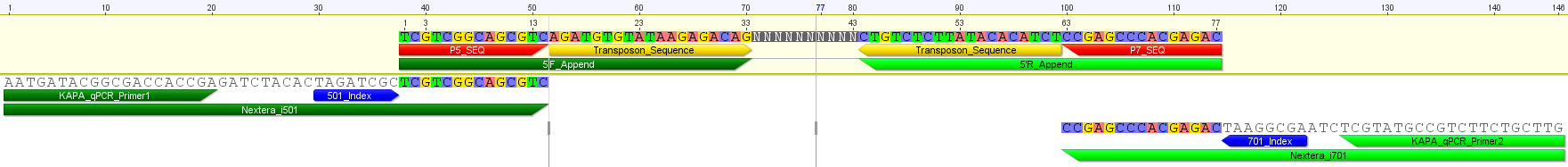
Quantify for sequencing
2. Analyze sequencing output

Unzip files

Merge reads

Align reads to reference Miseq_SeqPrep_manager.pl

Change to BAM format

Pileup reads for counts

Analyze for ratios analyze_ILLING_pileup.pl

1. Call putants from analysis
2. HRM and sequence putants

|  | P1 | P2 | P3 | P4 | … | PN |  |  |
| --- | --- | --- | --- | --- | --- | --- | --- | --- |
| Frag1 |  |  |  |  |  |  |  |  |
| Frag2 |  |  |  |  |  |  |  |  |
| … |  |  |  |  |  |  |  |  |
| FragN |  |  |  |  |  |  |  |  |
|  |  |  |  |  |  |  |  |  |
|  | S1 | S2 | S3 | S4 | … | SN |  | Final |

1. **DNA library and pooling**
   1. Genomic DNA of individual fish has been normalized to 10ng/ul. The Moens Lab library contains 9024 fish. Each fish has been pooled into 6Xpools on 96-well plates. This makes 1504 6Xpools, which fills 16 96-well plates (the last plate containing 64 6Xpools). These 6Xpools will be used to track the mutation carrier after each screening.


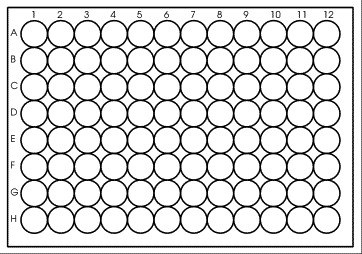

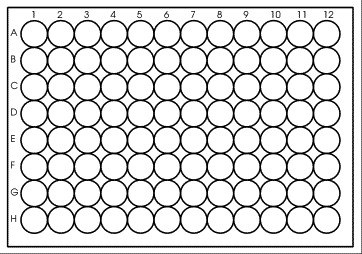

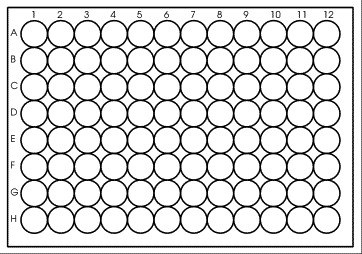

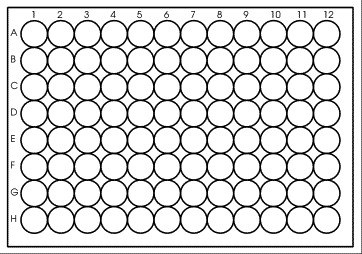

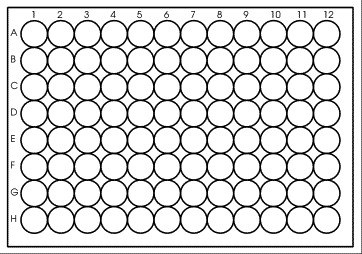

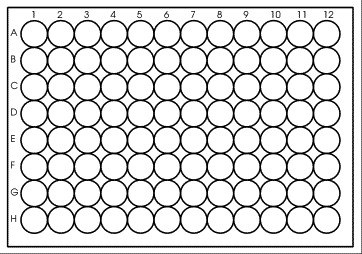


94 96-well plates gDNA library, 1 fish each well

16 6xPool plates, 6 fish in each well


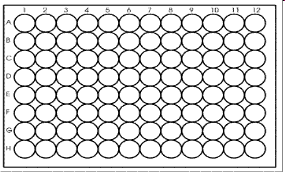

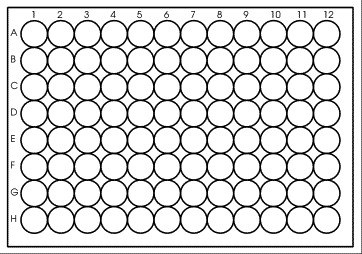

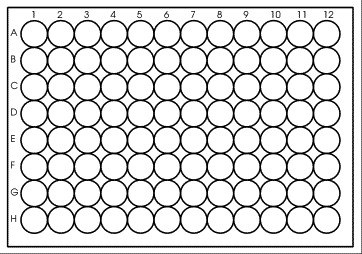

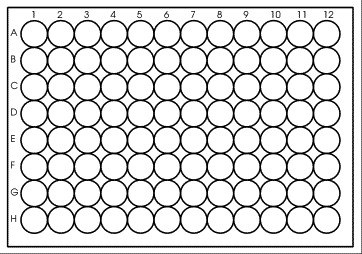


- 1. Each half of every 6Xpool plate (48 6Xpools) has been pooled again into one screening pool. 9024 fish then are pooled into a total 32 screening pools (each containing 288 fish with the last two pools containing 32 6Xpools or 192 fish). Each of these screening pools will be barcoded with one pair of Illumina Nextera indices in the final step.

32 screening pools (288 fish in each)

1. **Target selection and primer design**
   1. We are going to use the 250bp paired-end sequencing on the Illumina Miseq system. The target fragment must be between 210-270bp. Because the first step of sequencing data analysis is merging the paired-end sequences, any sequence that cannot be merged will be discarded. If the target fragment is 10bp larger then 250bp, 10bp sequence will be discarded from both ends causing a net 20bp loss. Since 20bp on both ends are primer sequence, we can afford to lose a net 40bp making the upper bound of the target size to be 270bp.) Illumina has updated the Miseq to a 600 reaction kit which is capable of 300bp paired-end sequencing. If the 300bp-Miseq system is used, the fragment size can be 260-320bp.
   2. For each target gene, multiple exons can be selected as target fragments according to the gene structure and CODDLE analysis result. For a large exon, use multiple fragments to cover the coding sequence.
   3. The current Miseq run can generate ~8Gb data from a good quality run, while data above Q30 may reach ~70% (giving us 5.5Gb). The total set screening size should be determined by the library size. For example, in the Moens lab library, we have 9024 Het fish. If we want every nucleotide of every fragment to be read at least 20 times from both directions, our calculation is:

$$Screening Size= \frac{5.5Gbp}{9024 fish \times2 alleles\times2 directions \times20 coverage}=7618bp$$

Our current set screening size is ~6kb, which contains 22-25 fragments.

- 1. Primers should be 18-26bp with overhangs as below:

Append to 5’ end of forward PCR primer:

5’ TCGTCGGCAGCGTCAGATGTGTATAAGAGACAG-[specific sequence]

Append to 5’ end of reverse PCR primers:

5’ GTCTCGTGGGCTCGGAGATGTGTATAAGAGACAG-[specific sequence]

- 1. Elute the primers in TE, and make each paired primer mix in H2O at a final concentration of 5uM each.
  2. Do a primer test for each pair of primers to determine the Tm.

|  | Temp | Time (sec) |
| --- | --- | --- |
| 1 | 98 | 60 |
| 2 | 98 | 10 |
| 3 | 50-70 | 30 |
| 4 | 72 | 40 |
|  | Go to 2 | 30x |
| 5 | 72 | 60 |
| 6 | 10 | hold |

| Component | Volume (uL) |
| --- | --- |
| H2O | 3.75 |
| 2x Phusion Mix | 5 |
| Primer Mix (5uM ea) | 0.25 |
| 10ng/uL gDNA | 1 |
| **Total** | **10** |

If you see a strong primer amplicon (~100bp), increase gDNA amount to 1.2-1.5uL, and/or reduce primer mix to 0.15-0.2uL per 10uL reaction. This primer test tells us:

1. The best annealing temperature for each fragment
2. How much gDNA is needed for each reaction
3. How much primer mix is needed for each reaction
4. For some highly amplified fragments, 28 cycles should be used in the target specific PCR, while faint fragments should use 32 cycles.

An example of a primer test:


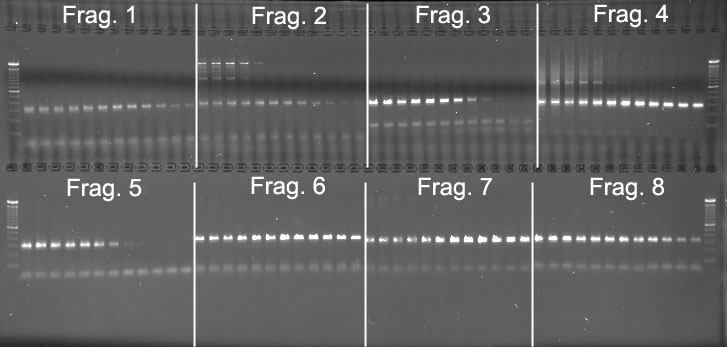


Here are 8 fragments tested using the above conditions. Each fragment is arrayed from 50-70° in 12 wells from left to right. We used Invitrogen 100bp DNA ladder.

According to the primer test results, the best annealing temperature should be picked for each fragment – strongest amplification without unspecific bands. In the above gel, Frag1 and Frag2 show slightly weaker amplification than the others. For these two fragments, template gDNA should be increased to 1.2uL per 10uL reaction. Frag3 has a strong band around 100bp, therefore the primer mix amount should be decreased to 0.2uL per 10uL reaction

1. **1^st^ PCR (Target specific PCR)**
   1. Each target fragment should be amplified with gDNA from the screening pools, so that the total target specific PCR reactions should be 32 x # target fragments.

|  | Temp | Time (sec) |
| --- | --- | --- |
| 1 | 98 | 60 |
| 2 | 98 | 10 |
| 3 | Tm | 30 |
| 4 | 72 | 40 |
|  | Go to 2 | 28-32x |
| 5 | 72 | 60 |
| 6 | 10 | hold |

| Component | Volume (uL) |
| --- | --- |
| H2O | to 20 |
| 2x Phusion Mix | 10 |
| Primer Mix (5uM ea) | 0.3-0.5 |
| 10ng/uL gDNA | 2-3 |
| **Total** | **20** |

1. For each target fragment, pick 4 pools to run a gel. Take 8uL product and run a 1.5% agarose 1X SYBR Safe gel. Quantify the concentration of each band using gel analysis software. Use the average concentration across these four pools as the concentration of the target fragment.


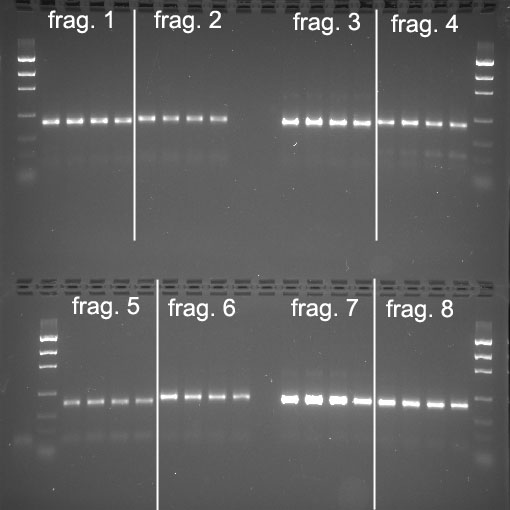
It would be ideal if all pools of all target fragments were tested on gels. We only run 4 pools to save time and expense. For each set, the 4 pools should be selected randomly, but within the set the 4 tested pools should be the same between fragments.

Here, 2uL Invitrogen Low DNA Mass Ladder was loaded along with 8 target fragments.

The average concentration of each fragment is calculated according the band intensity quantified by the gel analysis software. In our experience, it can be in the range of 5-50ng/uL.

1. 50ng from each fragment’s 1^st^ PCR product is combined by screening pool to get 32 pools. This pooled PCR product contains all target fragments amplified from this screening pool.
2. Pooled PCR product is cleaned using the ZYMO columns, and eluted in 25uL H2O. Take 4uL of this clean product from each of the 32 pools and run a 1.5% agarose 1X SYBR Safe gel. Quantify the harvest – it should be above 75%.
3. **2^nd^ PCR (Illumina indices PCR) and Pool**
4. Remember that the template DNA for the 2^nd^ PCR is the cleaned product from the 1^st^ PCR. Template amount should be 50ng in a 50uL reaction.
5. In the 2^nd^ PCR, two sets of primers will be used. The first pair comes from the Illumina Nextera Index Kit. According to the number of pools, certain indices combinations should be decided. Eeach screening pool should be barcoded using a specific pair of indices. The second pair of primers are the OutSide primer mix, which helps to improve the efficiency of full-length products.

OutSide primer forward: AATGATACGGCGACCACCGA

OutSide primer reverse: CAAGCAGAAGACGGCATACGA

1. The 2^nd^ PCR adds the Illumina indices and the sequence priming sites.

|  | Temp | Time (sec) |
| --- | --- | --- |
| 1 | 95 | 180 |
| 2 | 98 | 20 |
| 3 | 61 | 20 |
| 4 | 72 | 60 |
|  | Go to 2 | 5x |
| 5 | 72 | 180 |
| 6 | 10 | hold |

| Component | Volume (uL) | |
| --- | --- | --- |
| H2O | to 50 | |
| 2x KAPA Mix | 25 | |
| OutSide primers (5uM ea) | 5 | |
| Nextera i7 primer | 2.5 | |
| Nextera i5 primer | 2.5 | |
| Cleaned 1^st^ PCR product | ~50ng | |
| **Total** | **50** |  |


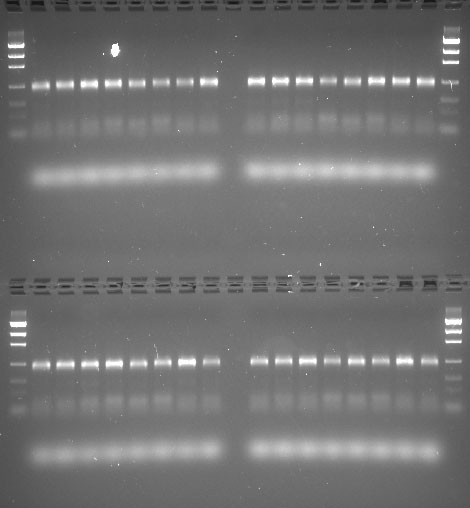


1. 2^nd^ PCR product should be target fragment size + 136bp. Take 4uL of this product from each of the 32 pools and run a 1.5% agarose 1X SYBR Safe gel.
2. 2^nd^ PCR product is cleaned with ZYMO columns, and eluted in 30uL H2O. The concentration of the cleaned product is checked using Invitrogen Quant-iT dsDNA assay kit or any other spectrophotometer system.
3. 50ng cleaned 2^nd^ PCR product is combined from each pool for the final loading sample.
4. **Quantify for sequencing**
   1. Follow the instructions on the KAPA Library Quantification kit. We typically have successful amplification and quantification of dilution of 1:100, 1:1K, 1:10K, 1:100K, 1:1M, and 1:10M. Quantify each dilution of the library in triplicate. Use this value as the pM amount of the sample for loading on the Miseq machine. For example, we load 15pM, but this VERY much depends on the machine and library prep. It will be better to start at a low concentration than to overload the machine – go with what the MiSeq operator recommends.
   2. We typically see a cluster density of well above 900K/mm^2^ with over 1000K/mm^2^ being a very good run. Also, the >Q30 is usually about 80%.
5. **Analyze sequencing output**
   1. See list of supplemental files as attached. One of the supplemental files you will want is a list of all the mutations that could possibly create a stop codon. The script “STOP_FINDER.R” contains a small program that will analyze a genbank file along with your target fragment primers and print out a list of these potential stops mutations. R can be downloaded from <http://www.r-project.org/>.

Open “STOP_FINDER.R” in R or RStudio and source the file.

**>getwd()**

This will print the working directory.

**>setwd("C:/...")**

This will set the working directory. Set it to the folder where the genbank files live. This will also be the output directory.

**>miseq_stop_finder(a,b,c,d)**

a = "genbank_filename"

b = "Forward primer sequence"

c = "reverse primer sequence"

d = "exon number, or any text to include in the output filename. Don't use '.' here, it will confuse the filenaming"

Output will be in 'a'_genbank_exon'd'_potential_stops.txt

Once all of the target fragments have had a potential stops file created, list them all into one file separated by “Locating name_amplicon” as the .stops file using linux EOL.

- 1. Software you will need access to: novoalign, samtools, perl, gunzip (these are all standard on any university server), and SeqPrep (which you need to download and install -- maybe ask someone with more experience)

When loading the sequencer, the barcodes are assigned a name (in this case, our pool name) and when the sequencing is done, the machine will demultiplex -- all the sequences are stripped of the adapter sequences, then deposited into different files based on the adapter sequence. Off the sequencer, you will get a variety of files that are your raw zipped fastq files. They will look like this:

00A_S1_L001_R1_001.fastq.gz

00A_S1_L001_R2_001.fastq.gz

These correspond to pool 00A read 1 and read 2.

The supplemental file you need at this step is just the fasta file that has all the amplicon sequences. In the amplicon name, do not use "." or more than one "_". Fasta format means:

>XX_exY

sequence_without_spaces_or_newlines

>name_amplicon2

sequence2_without_spaces_or_newlines

- 1. Once all the supplemental files and the fastq.gz files are in the same directory, we can start. The first thing to do is to unzip all the files.

**$ gunzip *.fastq.gz**

Now all the files will look like this:

00A_S1_L001_R1_001.fastq

00A_S1_L001_R2_001.fastq

Now, the aligner needs to know what to align the reads to and what positions all the nucleotides are at. So we need to make an index file of the reference sequence.

**$ novoindex refseq_ILL05.index refseq_ILL05.fasta**

//The first filename is the generated index file, and the second file is the fasta file. If novoindex is not on your PATH, type out the full path to novoindex.

**$ ~/../../app/novocraft/2.08/novoindex refseq_ILL05.index refseq_ILL05.fasta**

We will now run MiSeq_SeqPrep_manager.pl which needs to know all the filenames and paths of the various tools it needs to use in order to accomplish everything. This is a script that runs the SeqPrep program to merge the reads, then the merged reads are aligned to the reference sequence index using novoalign, then samtools is used to convert the alignment files into sam/bam format needed for the next step.

Changes you have to do once:

Line035 -- change the path of SeqPrep

//Eric has the option -n 0.97, I have used -n 1.0 as well

Line077 -- change the path of novoalign

Line106 -- change the path of samtools

Line111 -- change the path of samtools

Changes you have to do every set:

Line004 -- change this to your .index file

Line005 -- change this to the first part of the .fasta file

Line060 -- same as Line05

Now that the MiSeq_SeqPrep_manager.pl script knows where all the files and software is located it will take care of the merging and aligning. This will take a while and will generate the rest of the files.

**$ perl MiSeq_SeqPrep_manager.pl**

// If you want to save the output, which contains metrics like how many pairs of reads in each pool, the number of merged pairs, and the alignment counts

**$ perl MiSeq_SeqPrep_manager.pl >output05B.txt 2>&1**

- 1. Now all the reads are aligned to the reference sequences and all that data is stored in the sorted bam files. Now we use samtools pileup function to count reads and nucleotides at each position -- this will give us the SNP ratios necessary to make mutation calls. Samtools requires an index of the reference sequence that is different than the novoalign style index. Then we run mpileup on all of the sorted bam files.

**$ samtools faidx refseq_ILL05.fasta**

**$ samtools mpileup -BQ40 -d500000 -D -f refseq_ILL05.fasta *..bam_sorted.bam > ILL05B_fullpileup.txt**

Now, the pileup of all the reads for all the pools is stored in the fullpileup.txt file. Now we are going to use the analyze_ILLING_pileup.pl script to do the analysis and get the mutation ratios. This script requires two supplemental files -- a copy of the refseq_ILL05.fasta but as a .txt file, as well as the ILL05.stops file. The .stops file is a list of the stops generated by the stop_finder program separated by "Locating XX_exY" and with unix style EOL characters. Also, in order for the mpileup to act on all of our pools we need to create a list of all the bam files it needs to analyze

**$ ls *..bam_sorted.bam >> ILL05B_bam.txt**

We also need to let analyze_ILLING_pileup.pl know what all these filenames are.

Line009 -- change filename to ILL05B_bam.txt

Line027 -- change filename to refseq_ILL05.txt

Line047 -- change filename to ILL05.stops

Now we can run the final analysis of the pileup. And we want to pipe the output to a new file instead of to the terminal.

**$ perl analyze_ILLING_pileup.pl ILL05B_fullpileup.txt > ILL05B_DATA.txt**

The output is in a tab delimited txt file that can be opened and analyzed in excel.

1. **Call putants from analysis**
   1. Open the txt file in excel. The first row will be the names of all the pools. Following this are the target fragments where each nucleotide has three alternate calls listed after it. The easiest way to look at this file is to separate the fragments onto different sheets of excel and freezing the top row with the pool labels.
   2. The “ratio” that is printed in various locations is the ratio of this alternate base pair to the reference base pair in the total reads covering this locus. Due to our pooling strategy, each of these screening pools has 288 fish or 576 alleles. If everything is ideal, we should see a single heterozygous fish’ mutant allele at a ratio of 1:576. This is not always true, so we allow for 50% error due to PCR, pooling, sequencing, etc. So we are looking for ratios around greater than 1:864; however, ratios greater than 1:16 are usually polymorphisms and not ENU induced mutations.
   3. Scan through the file for good ratios, especially if they occur at the potential stop sites. Usually if the call has occurred in more than two pools, it is noise and not real. This is something that you will have to work out based on the results and sequencing depth of your run.
   4. Mark all the calls that could potentially be real and create a new file with the position of all these calls for each fragment, the pool they reside in, as well as the ratio, and type of mutation.
2. **HRM and sequence putants**
   1. We use High Resolution Melt Analysis (HRM) to detect the real mutations carriers from our screening pools. Every putant call corresponds to one screening pool, which contains 288 fish or 576 alleles, and occupies one half 96well plate of 6xPool (48 wells). We use this half plate of 6xPool fish to detect the mutation carrier by HRM, then the corresponding 6 individuals are screened by HRM and the single mutation carrier is found. We then confirm by ABI sequencing.
   2. Each pair of HRM primers should amplify a small fragment containing your putant (or putants) with a size restricted to 60-150bp. Since we are screening our mutation carrier in a 6xPool, we are trying to detect 1 mutant allele among 11 WT alleles; therefore, an amplicon that is too large would disturb the subtle difference between the target mutation carrier and the non-target pools.

C/G to T/A mutations are more easily detected due to the change in hydrogen bonds. In this case, a larger (100-150bp) amplicon can be selected. T/A to A/T give relatively subtle changes of melting temperature difference from wildtype. In this case, a smaller (60-100bp) amplicon should be selected. G/C to C/G mutations are very rare in ENU mutagenesis. HRM primers should be tested with a gradient program to decide the best Tm.

- 1. HRM reactions are done in an rtPCR machine with a camera that detects fluorescence. The machine basically detects the small difference in melting temperature that can be attributed to a mutation between the various wells on the plate. The resolution of the BioRad CFX Connect is 0.1°. The PCR has no extension step because the amplicon size is quite small. After the PCR is completed, the melt starts. The machine will go from 65° to 90°. At every 0.1° increment, the machine holds that temperature for 5seconds in which time it takes a fluorescent measure.

|  | Temp | Time (sec) |
| --- | --- | --- |
| 1 | 95 | 180 |
| 2 | 95 | 10 |
| 3 | Tm | 40 |
|  | Go to 2 | 40x |
| 4 | 65 to | Holding 5 |
| 5 | 90 | by 0.1° |

| Component | Volume (uL) |
| --- | --- |
| H2O | to 20 |
| 2x HRM Mix | 10 |
| Primer Mix (5uM ea) | 1 |
| gDNA of 6xPool | 10ng |
| **Total** | **20** |

- 1. Examples of 6xPool HRM (each line represents one 6xPool)

CtoT mutation TtoA mutation


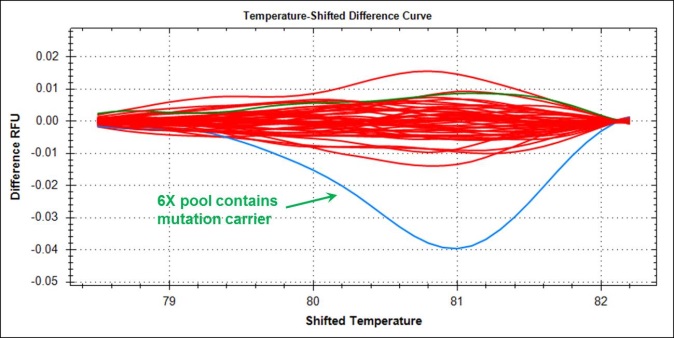

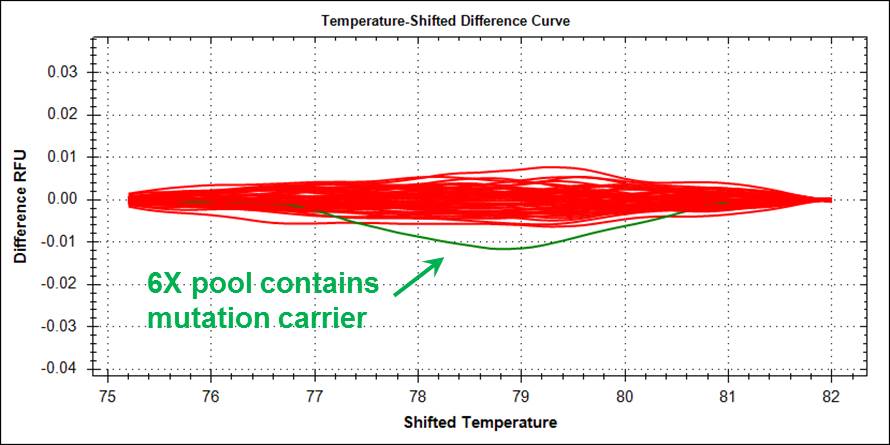


- 1. Examples of 6 individual HRM (each line represents one individual)

CtoT mutation TtoA mutation


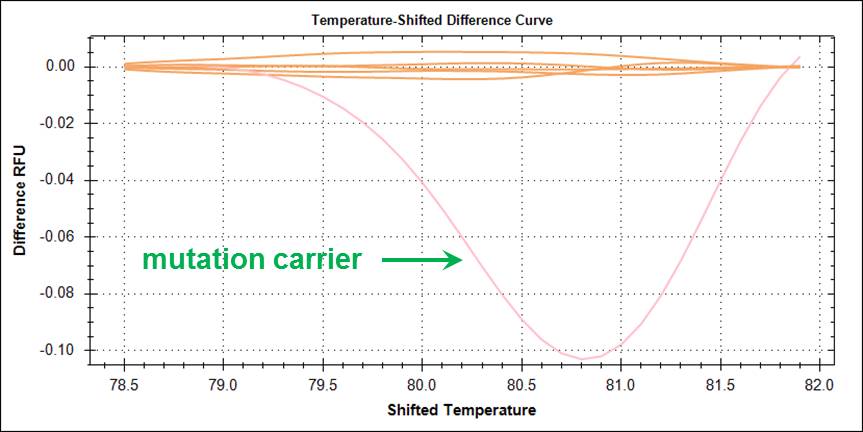

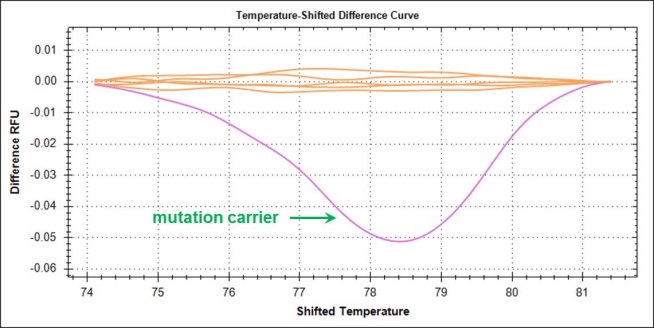


- 1. Putant carriers can be amplified with 1^st^ PCR primers and sequenced with sequencing primer to confirm the real mutation.

Miseq Seq F: GCAGCGTCAGATGTGTATA

Miseq Seq R: GGCTCGGAGATGTGTATAA

1. **Product Numbers**

Primer test, 1^st^ PCR

Phusion HF Master Mix NEB M0531L

Gels

SYBR Safe DNA Gel Stain Invitrogen S33102

1^st^ + 2^nd^ PCR Cleanup

ZYMO DNA Clean and Concentrator 5 ZYMO D4014

2^nd^ PCR

KAPA HiFi HotStart Ready Mix KAPA KK2602

Nextera Index Kit 96 Indices Illumina FC-121-1012

Quantify final library

KAPA llumina Library Quantify KAPA KK4824

Sequencing

MiSeq Reagent Kit V2, 500cycles Illumina MS-102-2003

HRM

Biorad Precision Melt Supermix BioRad 172-5112

Biorad HardShell ThinWall 96well BioRad HSP-9601

Biorad Microseal 'B' Adhesive Seals BioRad MSB-1001
