# Supplementary figures and images for: Rapid identification and recovery of ENU-induced mutations with next-generation sequencing and Paired-End Low-Error analysis
Source: BMC Genomics. 2015 Feb 14;16(1):83. doi: 10.1186/s12864-015-1263-4 (PMC4457992; doi:10.1186/s12864-015-1263-4)

Difference Curve

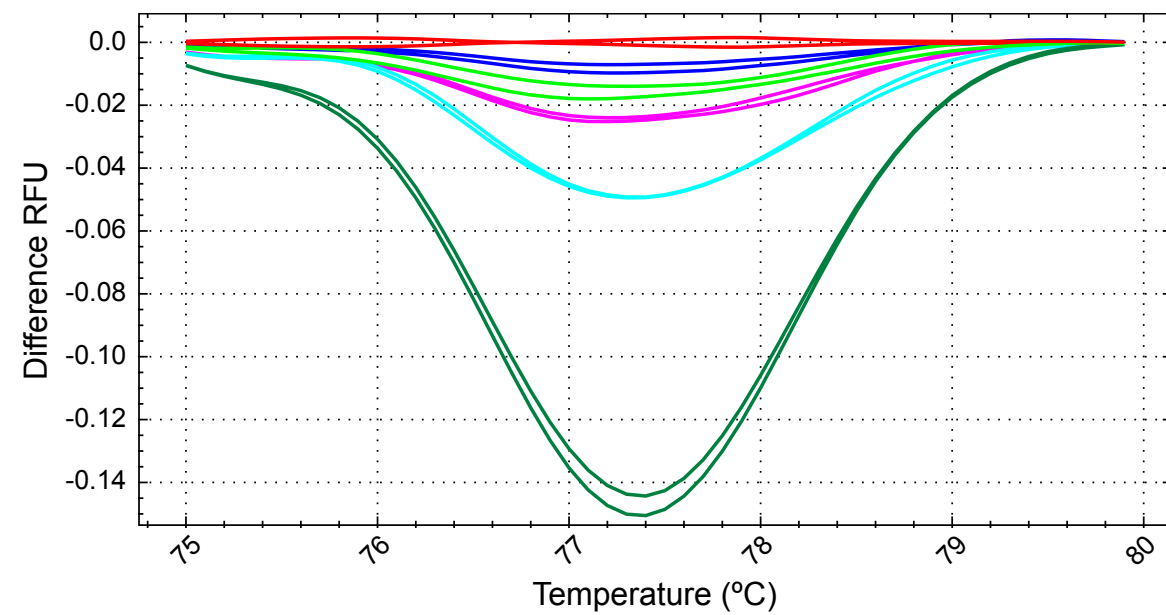

Supplement: Additional file 7: — HRM Detection of Mutant Alleles at Various Mut:WT Ratios. High Resolution Melt (HRM) Analysis Detection of Mutant Alleles at Various Mut:WT ratios. The mutant allele being tested here is inka1af h326, a C > T mutation resulting in a nonsense mutation, R120X. HRM melt curves corresponding to wildtype (red; 0 mutant alleles), 12 animals (dark blue; 1 mutant allele in 24 alleles), 6 animals (light green; 1 in 12 alleles), 4 animals (pink; 1 in 8 alleles), 3 animals (light blue; 1 in 6 alleles), 1 animal (dark green; 1 in 2 alleles). Although the deflection due to the mutant allele is much more dramatic at lower ratios, it can still be detected at a 1:12 ratio (dark blue line). [file 12864_2015_1263_MOESM7_ESM.pdf]
